# Improving education and coping of scoliosis patients undergoing surgery, and their families, using e-health

**DOI:** 10.1007/s11832-016-0772-2

**Published:** 2016-10-06

**Authors:** Magdalena Lysenko, Peggy Law, James Jarvis, James G. Wright

**Affiliations:** 1 Division of Orthopedic Surgery, The Hospital for Sick Children (SickKids), 555 University Ave., Toronto, ON M5G 1X8 Canada; 2 Faculty of Orthopaedic Surgery, University of Toronto, Toronto, ON Canada; 3 Children’s Hospital of Eastern Ontario, Ottawa, ON Canada

**Keywords:** Website, Scoliosis, AIS, E-health, Scoliosis surgery

## Abstract

**Purpose:**

Healthcare providers have limited time to spend with scoliosis patients who are considering surgery and their families. The purpose of this study was to evaluate an e-health strategy to increase knowledge and coping in patients with scoliosis who are surgical candidates and their families.

**Methods:**

We enrolled patients with scoliosis who were candidates for surgery and their families. Patients and their families completed the scoliosis knowledge questionnaire, meaning of illness questionnaire, social support and coping questionnaires before and after access to a comprehensive evidence-based scoliosis website (http://www.aboutkidshealth.ca/scoliosis).

**Results:**

Seventy-four patients and 71 parents completed the evaluation. While both patients and parents improved their knowledge of scoliosis (*p* = 0.001 and *p* = 0.003, respectively), the scores of patients were consistently lower than those of the parents both before and after website use (*p* = 0.0001). Only parents demonstrated a change in the meaning of illness questionnaire, with a small increase in the negative attitude towards illness and a small decrease in the positive attitude towards illness (*p* = 0002 and *p* = 0.01, respectively). Of the 12 coping methods examined on the Adolescent Coping Orientation for Problem Experiences (A-COPE) instrument, patients were slightly more likely than parents to use relaxing and solving family problems as tools to cope following website access (*p* = 0.02 and *p* = 0.09, respectively). Parents demonstrated no significant changes in the four methods of coping on the Coping Health Inventory for Parents (CHIP) after website exposure. While the majority of patients and parents reported receiving sufficient support, over half of the patients indicated a need for more support in social participation.

**Conclusion:**

An evidence-based website increased the knowledge of patients and parents but simply providing access to the website had minimal impact on their coping and perceptions of social support. The website, however, provides users with the opportunity to absorb vital information about scoliosis across several media.

## Introduction

E-health, defined as the application of the internet and related technologies in health care to improve quality of care, holds great promise to address unmet educational needs of families seeking medical information [1–7]. Computer-based education has the advantage of providing standardized information occurring at a manageable pace for the family and patient, and at a time when they are potentially better prepared to comprehend the diagnosis and medical procedures. In addition, it is hoped that better understanding would in turn enable patients and parents to be more involved in the decision-making process and thereby enhance the therapeutic relationship.

Approximately 10 % of adolescents with idiopathic scoliosis will progress to a state where surgical intervention becomes a consideration [8, 9]. The goals of surgery are to correct the spinal deformity and minimize further progression [10]. Although it is the most common paediatric orthopaedic procedure performed in the United States, correction of scoliosis is a major surgery [11, 12].

Parents have a special obligation to prepare their children for the procedure and hospital experience [13]. Patients with little knowledge of their disease are five times more likely to feel insecure and helpless [14]. This decisional distress has been correlated not only with making parents less capable of providing support and reducing stress in their children but is also associated with post-traumatic stress disorder in children [15, 16]. Previous research has shown that the information needs of families are often unmet [17–19]. Families and patients with scoliosis, compared with those diagnosed with other orthopaedic conditions, use the internet nearly twice as often to understand their diagnosis [20]. While there is a large volume of information available online, the information varies enormously in quality, leading to confusion and misinformation [21]. Websites often lack evidence-based information, have poor usability, and do not focus on family support. In addition, several websites integrate product endorsement, thereby introducing a potentially confusing mix of patient/parent information with industry promotion.

The purpose of this study was to evaluate an e-health strategy to increase knowledge and coping in patients with scoliosis undergoing surgery and their families.

## Methods

### Development of website

The development of the http://www.aboutkidshealth.ca/scoliosis website involved a needs assessment, qualitative and quantitative analyses of current online resources, and development of evidence-based content.

In previous research we evaluated the needs of patients, families and healthcare providers for a website to provide information and support for adolescents considering surgery for scoliosis, and their families [22]. Healthcare providers indicated that the content, format, layout and graphics should reflect users’ needs, be professionally moderated and have an interactive support component where users can access information from peers and healthcare providers. Furthermore, a website ideally should provide information for a variety of surgical candidates taking into consideration age, gender, reading level and geographic location of users [22]. Concerns raised by patients with adolescent idiopathic scoliosis (AIS) considering surgery included recovery at home and in the hospital (side-effects, hospital stay, recovery time), post-surgical appearance, emotional impact of surgery and coping, intrusion of surgery and recovery of daily activities, impact of surgery on school, peer relationships and other social interactions (possible over-reactions of others), decision-making about surgery, being in the operating room, and future concerns (i.e. pregnancy, employment, future discomfort and problems) [23].

Based on the findings from the needs assessment, a wireframe was developed to present the preliminary organization and navigation through the website. The website content was based on a literature review which addressed a broad base of scoliosis outcomes, both treated and non-treated, and specifically focused on patients’ and families’ informational needs. Identified studies were graded using levels of evidence [24, 25] and “gray areas” were acknowledged, whether due to contradictory or conflicting evidence [26]. Site modules addressed multiple topics including diagnostics, treatment options, outcomes of surgery, course of surgery and post-surgical care, all organized with intuitive navigational architecture. Focus groups (patients, parents and healthcare professionals) provided feedback during website development.

### Participants for website evaluation

Patients with AIS between the ages of 10 and 18 years, along with their parents or legal guardians (hereby referred to as parents), were recruited from The Hospital for Sick Children and Children's Hospital of Eastern Ontario. None had received any previous spinal surgery. All patients had a curve size within or approaching the range of surgical consideration (Cobb angle > 40°). Access to internet either from home, school, or public library and fluency in English were required.

The sample size calculation was based on effect sizes expressed in standard deviations. Non-randomized designs often overestimate the true effect size and thus we chose a “large” effect size because a smaller effect size in a non-randomized design would not be clinically significant [27]. Based on the conservative assumption that the pre- and post-measurements would have a correlation of 0.5, a sample size of 102 pairs of patients and parents would have an 80 % power to achieve statistical significance, if the true difference between pre- and post-means demonstrated an effect size of 0.68 for the primary outcomes of improvement in coping and 0.83 for the other outcomes.

Participants provided consent and completed baseline questionnaires online prior to website access and 6 weeks thereafter.

### Questionnaires

The *Scoliosis Knowledge Questionnaire*, a 14-item, open-ended, short-answer questionnaire developed for this study has test–retest reliability (intra-class correlation coefficients) of 0.96 and 0.64 for parents and adolescents, respectively [27]. Known group construct validity was confirmed by statistically significant differences between pre-operative braced and post-operative scoliosis patients (*p* = 0.01). The instrument has been tested on both adolescent and adult populations.

The *Meaning of Illness Questionnaire (*MIQ), a 30-item instrument measuring cognitive appraisal of illness and associated stresses [28], has five factors: (1) impact of illness; (2) type of stress (negative attitude of harm, loss, threat and functional context (prognosis); (3) degree of stress or change in commitments/secondary appraisal of coping resources; (4) positive attitude (challenge, hope, motivation and control); and (5) expectancy and recurrence. The test–retest reliability is satisfactory (kappa = 0.45–0.99), internal consistency has been demonstrated and the measure has fair to moderate validity. The MIQ has been used in both adolescent and adult populations.

The *Adolescent Coping Orientation for Problem Experience (A*-*COPE),* a 54-item instrument measuring behaviors adolescents use in managing difficult situations and life changes, is organized according to 12 coping patterns: (1) ventilating feelings; (2) seeking diversions; (3) developing self-reliance and optimism; (4) developing social support; (5) solving family problems; (6) avoiding problems; (7) seeking spiritual support; (8) investing in close friends; (9) seeking professional support; (10) engaging in demanding activity; (11) being humorous; and (12) relaxing [29]. The A-COPE has internal consistency (alpha ranging from 0.50 to 0.75) and test–retest correlation of 0.83.

The *Coping Health Inventory for Parents (*CHIP), a 45-item multi-dimensional measure for parents’ perceived coping with childhood chronic illness, has internal consistency reliability (Cronbach’s alpha) ranging from 0.71 to 0.99. Only four questions regarding coping behaviours were examined: (1) talking over personal feelings and concerns with spouse; (2) believing that my child(ren) will get better; (3) talking with the doctor about my concerns about my child(ren) with the medical condition; (4) believing that the medical centre or hospital has my family’s best interest in mind [29].

The *Arizona Social Support Interview Schedule,* a semi-structured interview, examines six different types of support: (1) shared feelings, (2) material aid, (3) advice, (4) positive feedback, (5) physical assistance and (6) social participation [30]. This questionnaire was revised slightly for the present study in that all questions were completed online and only the need for support and whether the amount of support provided was adequate to the person’s needs were analyzed.

### Statistical analysis

Each questionnaire was scored before and after website access. The data from the patients and parents was analyzed separately. One-sample, two-sided *t* tests were performed on the differences between pre- and post-measurement.

## Results

We enrolled 136 patients and 126 parents. A total of 74 patients (90.5 % female) and 71 parents (84.5 % female), from a variety of ethnicities including Caucasian, African, Middle Eastern and Asian, completed both sets of questionnaires. The mean age of the responders was 14.3 ± 1.9 years (patients, range 10–18 years) and 45.9 + 6.1 years (parents, range 31–60 years). The majority of parents had at minimum a high-school education and over 60 % reported an annual income of over $80,000 (CAD). Many patients (75.7 %) were able to identify the type and size of their curve. At the time of their study participation, 37.8 % of patients were on the waiting list for surgery (mean 2.8 ± 3.0 months). Over 95 % of the patients and parents reported regular use of the internet. Of this group, while 83.3 % of patients and 91.2 % of parents had previously searched for information on scoliosis, only 13.5 and 18 %, respectively, had found the information to be very helpful (Table 1).

**Table 1:** Demographic information for patients and parent

Descriptive	Patients (*n* = 74)	Parents (*n* = 71)
Sex		
Female	67 (90.5 %)	60 (84.5 %)
Male	7 (9.5 %)	11 (15.5 %)
Age (years)	14.3 ± 1.9	45.9 ± 6.1
Do you know your curve (yes)?	56 (75.7 %)	
Curve information		
Upper thoracic	42 (56.7 %)	
Upper thoracic curve size (degrees)	52.5 ± 15.4	
Thoracic	16 (21.6 %)	
Upper thoracic curve size (degrees)	51.9 ± 22.0	
Lumbar	38 (51.4 %)	
Lumbar curve size (degrees)	44.2 ± 14.9	
Brace		
Brace wear (yes)	27 (36.5 %)	
Duration of brace wear (months)	24.2 ± 18.0	
Surgery		
Surgical waiting list (yes)	28 (37.8 %)	
Duration on surgical waiting list (months)	2.8 ± 3.0	
Education		
Elementary	72 (97.3 %)	1 (1.4 %)
High school	2 (2.7 %)	67 (94.4 %)
College/university		3 (4.2 %)
Income (CAD)		
$0–$24,999		2 (2.8 %)
$25,000–$49,999		8 (11.3 %)
$50,000–$79,999		17 (23.9 %)
$80,000–$124,999		22 (31.0 %)
$125,000–$149,999		6 (8.5 %)
$150,000 +		16 (22.5 %)
Number of children under 18 in the home		
1		26 (36.6 %)
2		35 (49.3 %)
3		4 (5.6 %)
4		5 (7.0 %)
5		1 (1.4 %)
Number of parents in the home		
Single-parent household		19 (26.8 %)
Dual-parent household		52 (73.2 %)
Race		
Arab/West Asian	2 (2.7 %)	1 (1.4 %)
Black	4 (5.4 %)	3 (4.2 %)
Chinese	5 (6.8 %)	5 (7.0 %)
Filipino	1 (1.4 %)	0 (0 %)
Japanese	0 (0 %)	1 (1.4 %)
Korean	1 (1.4 %)	0 (0 %)
South Asian	3 (4.1 %)	1 (1.4 %)
South East Asian	1 (1.4 %)	0 (0 %)
White	54 (80.0 %)	59 (83.1 %)
Other	3 (4.1 %)	1 (1.4 %)
First language spoken		
English	63 (85.1 %)	51 (71.8 %)
French	5 (6.8 %)	5 (7.0 %)
Chinese	4 (5.4 %)	4 (5.6 %)
Urdu	0 (0 %)	3 (4.2 %)
Italian	0 (0 %)	3 (4.2 %)
Other	2 (2.7 %)	5 (7.0 %)
Use of online sources		
Email	71 (96.0 %)	67 (95.7 %)
Instant messaging	54 (73.0 %)	21 (29.6 %)
Blog	6 (8.1 %)	2 (2.8 %)
Social networks	59 (79.7 %)	30 (42.3 %)
News	20 (27.0 %)	49 (69.0 %)
Leisure	13 (17.6 %)	49 (69.0 %)
Work/school	63 (85.1 %)	42 (59.2 %)
Banking	5 (6.8 %)	53 (74.7 %)
VOIP	14 (18.9 %)	15 (21.1 %)
Health information		
Search for health information	54 (73.0 %)	68 (95.8 %)
Search once/week	6 (11.1 %)	17 (25.0 %)
Search once/month	13 (24.1 %)	17 (25.0 %)
Search few/year	35 (64.8 %)	34 (50.0 %)
Search for scoliosis information	45 (83.3 %)	62 (91.2 %)
Was the information helpful?		
Not helpful	6 (8.1 %)	3 (4.2 %)
Somewhat helpful	30 (40.5 %)	46 (63.9 %)
Very helpful	10 (13.5 %)	13 (18.1 %)

An additional 62 patients and 55 parents completed the pre-website access but did not complete the post-website set of questionnaires, citing the following reasons: lack of time, technical problems and general disinterest. Parents who did not complete the study were more likely to use social networks (62.3 %) than parents who completed the study (42.3 %; *p* = 0.024). Patients who completed the study were more likely to use instant messaging (IM) (73 %) than patients who did not complete the study (54 %; *p* = 0.042). In addition, patients who completed the study were more likely to know their curve size (75.7 %) than those patients who did not complete the study (48.8 %; *p* = 0.004). No other differences existed for parents or patients when comparing those who did and did not complete the study.

### Questionnaires

#### Scoliosis Knowledge Questionnaire

While patients and parents improved their knowledge of scoliosis after exposure to our website (*p* = 0.001 and *p* = 0.003, respectively) (Tables 2, 3), the scores of patients were consistently lower than those of the parents both before and after website use (*p* = 0.0001).

**Table 2:** Patient responses to scoliosis knowledge questionnaire, meaning of illness questionnaire, and adolescent coping orientation for problem experiences

Questionnaire descriptive pre/post	Patients (*n* = 74)	95 % confidence interval difference	*p* value
Knowledge questionnaire			
Pre	7.8 ± 4.2		0.001*
Post	9.5 ± 4.6		
Meaning of illness questionnaire			
Impact of illness			0.89
Pre	3.97 ± 1.7		
Post	3.8 ± 1.4		
Types of stress, attitude of harm, loss, threat, functional context			
Pre	3.0 ± 1.2		0.58
Post	3.1 ± 1.4		
Degrees of stress, change in commitments, secondary appraisal			
Pre	4.1 ± 1.1		0.37
Post	4.3 ± 1.1		
Challenge, positive attitude, motivation, hope			
Pre	4.3 ± 0.8		0.32
Post	4.2 ± 0.8		
Non-anticipated vulnerability			
Pre	3.2 ± 1.1		0.98
Post	3.2 ± 1.1		
Adolescent coping orientation for problem experiences			
Ventilating feelings			
Pre	3.2 ± 0.5	−016 to 0.07	0.40
Post	3.2 ± 0.5		
Seeking diversions			
Pre	2.9 ± 0.6	−0.06 to 0.20	0.31
Post	2.8 ± 0.6		
Developing self-reliance and optimism			
Pre	3.2 ± 0.6	−0.18 to 0.14	0.80
Post	3.2 ± 0.7		
Developing social support			
Pre	3.2 ± 0.6	−0.08 to 0.18	0.41
Post	3.1 ± 0.6		
Solving family problems			
Pre	2.9 ± 0.7	−0.25 to 0.02	0.09*
Post	3.1 ± 0.7		
Avoiding problems			
Pre	4.3 ± 0.4	−0.03 to 0.14	0.18
Post	4.3 ± 0.4		
Seeking spiritual support			
Pre	1.7 ± 0.9	−0.11 to 0.13	0.88
Post	1.7 ± 0.9		
Investing in close friends			
Pre	3.0 ± 0.9	−0.26 to 0.24	0.91
Post	3.0 ± 0.9		
Seeking professional support			
Pre	1.4 ± 0.6	−0.08 to 0.14	0.54
Post	1.4 ± 0.6		
Engaging in demanding activity			
Pre	3.0 ± 0.9	−0.10 to 0.25	0.41
Post	3.0 ± 0.8		
Being humorous			
Pre	3.4 ± 1.0	−0.20 to 0.25	0.81
Post	3.4 ± 0.9		
Relaxing			
Pre	3.1 ± 0.5	−0.30 to 0.02	0.02*
Post	3.3 ± 0.6		

* Significant at *p* < 0.05

**Table 3:** Parent responses to scoliosis knowledge questionnaire, meaning of illness questionnaire, and coping health inventory for parents

Questionnaire descriptive pre/post	Parents (*n* = 71)	95 % confidence interval difference	*p* value
Knowledge questionnaire			
Pre	10.4 ± 5.0		0.0026*
Post	12.9 ± 5.6		
Meaning of illness questionnaire			
Impact of illness			
Pre	4.3 ± 1.7		0.69
Post	4.4 ± 1.5		
Types of stress, attitude of harm, loss, threat, functional context			
Pre	2.3 ± 1.4		0.0002*
Post	2.8 ± 1.4		
Degrees of stress, change in commitments, secondary appraisal			
Pre	4.2 ± 1.1		0.78
Post	4.3 ± 1.0		
Challenge, positive attitude, motivation, hope			
Pre	4.8 ± 0.7		0.01*
Post	4.4 ± 1.0		
Non-anticipated vulnerability			
Pre	3.1 ± 1.0		0.51
Post	3.2 ± 1.1		
Coping health inventory for parents			
Talking over personal feelings and concerns with spouse			
Pre	3.8 ± 2.9	−0.61 to 0.82	0.79
Post	3.7 ± 2.9		
Talking with other individuals/parents in my same situation			
Pre	2.6 ± 1.0	−0.69 to 0.18	0.25
Post	2.9 ± 1.4		
Talking with the doctor about my concerns about my child(ren) with the medical condition			
Pre	2.8 ± 1.6	−0.22 to 0.65	0.33
Post	2.6 ± 1.1		
Believing that the medical centre/hospital has my family’s best interest in mind			
Pre	2.7 ± 0.5	−0.09 to 0.31	0.26
Post	2.6 ± 0.7		

* Significant at *p* < 0.05

#### Meaning of Illness Questionnaire (MIQ)

For the five factors examining cognitive appraisal of the illness and the associated stresses, patients did not differ before and after website exposure. Parents showed a small increase in the negative attitude towards illness and a small decrease in the positive attitude towards illness (*p* = 0002 and *p* = 0.01, respectively) (Tables 2, 3).

#### Adolescent Coping Orientation for Problem Experiences (A-COPE)

Of the 12 coping methods examined, only one increased: patients were slightly more likely to use relaxing and solving family problems as tools to cope (*p* = 0.02 and *p* = 0.09, respectively) (Table 2).

#### Coping Health Inventory for Parents (CHIP)

There were no significant changes in the four methods of coping after website exposure for parents (Table 3).

#### Arizona Social Support Interview Schedule

While the majority of patients and parents, prior to website exposure, reported receiving sufficient support in different forms (Table 4), over half of the patients indicated a need for more support in the social participation aspect.

**Table 4:** Parent completion of the arizona social support interview schedule prior to website exposure

Social support needed/received; amount needed/received	Patients (*n* = 74)	Parents (*n* = 71)
Share feelings		
Needed the support		
Need a lot more	12 (16.2 %)	7 (9.9 %)
Needed a little more	26 (35.1 %)	17 (23.9 %)
Received enough	36 (48.7 %)	47 (66.2 %)
Received the support		
Not at all	7 (9.5 %)	8 (5.5 %)
A little bit	49 (66.2 %)	42 (59.1 %)
Quite a bit	18 (24.3 %)	21 (29.6 %)
Receive material aid		
Needed the support		
Need a lot more	3 (4.1 %)	2 (2.8 %)
Needed a little more	9 (12.2 %)	1 (1.4 %)
Received enough	63 (83.8 %)	68 (95.8 %)
Received the support		
Not at all	38 (51.4 %)	47 (66.2 %)
A little bit	34 (46.0 %)	19 (26.8 %)
Quite a bit	2 (2.7 %)	5 (7.0 %)
Receive advice		
Needed the support		
Need a lot more	6 (8.1 %)	5 (7.0 %)
Needed a little more	26 (35.1 %)	7 (9.9 %)
Received enough	42 (56.8 %)	59 (83.1 %)
Received the support		
Not at all	14 (18.9 %)	6 (8.5 %)
A little bit	47 (63.5 %)	49 (69.0 %)
Quite a bit	13 (17.6 %)	16 (22.5 %)
Receive positive feedback		
Needed the support		
Needed a lot more	6 (8.1 %)	8 (11.3 %)
Needed a little more	24 (32.4 %)	13 (18.3 %)
Received enough	44 (59.5 %)	50 (70.4 %)
Received the support		
Not at al	25 (33.8 %)	14 (19.7 %)
A little bit	38 (51.4 %)	43 (60.6 %)
Quite a bit	11 (14.9 %)	14 (19.7 %)
Receive physical assistance		
Needed the support		
Need a lot more	4 (5.4 %)	8 (11.3 %)
Needed a little more	23 (31.1 %)	19 (26.8 %)
Received enough	47 (63.5 %)	44 (62.0 %)
Received the support		
Not at all	15 (20.3 %)	13 (18.3 %)
A little bit	51 (68.9 %)	45 (63.4 %)
Quite a bit	8 (10.8 %)	13 (18.3 %)
Social participation		
Needed the support		
Need a lot more	11 (14.9 %)	15 (21.2 %)
Needed a little more	39 (52.7 %)	18 (25.4 %)
Received enough	24 (32.4 %)	38 (53.5 %)
Received the support		
Not at all	6 (8.1 %)	8 (11.3 %)
A little bit	45 (60.8 %)	40 (56.3 %)
Quite a bit	23 (31.1 %)	23 (32.4 %)

## Discussion

E-health, including web-based, interventions have the potential to significantly enhance patient and family-healthcare provider interaction [31]. Many individuals are anxious during a clinic visit, making it difficult to understand or retain the information provided [32]. In contrast, web-based information may be accessed whenever and however patients and families wish, allowing time to digest the information on their own schedule [33, 34]. Prior research has demonstrated that the majority of questions raised with healthcare providers in a busy orthopaedic clinic can be found online [35].

The internet is a frequent and positively rated source of health information [20]. Most patients and their families learn about scoliosis by searching the internet after their diagnosis. Unfortunately, available online resources are variable and may contain conflicting information, making it difficult to discern which information is trustworthy [20]. The impact of illness-related websites can be improved by providing full and in-depth information with the opportunity for interaction [36]. This principle guided the development of the new scoliosis website for families and patients. We consulted extensively with patients, families and healthcare providers before and during website development to meet the users’ needs.

An important outcome of the patient experience is healthcare knowledge. Accurate knowledge may help reduce decisional conflict and promote active participation with healthcare providers in their care [37, 38]. Families with accurate and complete information reportedly have less anxiety and stress, which in turn contributes to a better operative experience [18]. Parents with knowledge can also prepare their child for upcoming medical procedures with better recuperation [39]. Furthermore, patients who understand their illness are able to modify their behaviors and expectations in order to promote healing [40]. We found that within the 6-week period both the parent and patient groups showed a significant improvement in knowledge. Thus an evidence-based website increases health knowledge for patients and families.

Despite prior research demonstrating a relationship between knowledge of disease and anxiety and helplessness [14], the study demonstrated a small increase in stress for parents following website exposure. This may have been due to the fact that the surgical date for some of their children was approaching and/or they learned additional information that they were otherwise unaware of (e.g. potential complications). The results of this study are important because they question the role of an e-health strategy such as websites in improving outcomes beyond increasing health knowledge for patients and families. While the quality varies, the orthopaedic information on the internet continues to increase. Consistent with other studies, virtually all our patients and families accessed the internet to find more information about scoliosis. Perhaps different strategies such as asynchronous moderated websites, social media or internet direct communication will be needed to enhance coping (or other health outcomes).

The website provides users with the opportunity to absorb vital information about scoliosis across several media. If provided earlier on, at time of diagnosis or as part of pre-operative preparation seminars, perhaps it would have an even greater impact. This study has several potential limitations. First, the lack of improvement in areas beyond knowledge may have been because these patients already had strong support strategies in place. Parents reported that they received enough support in sharing their feelings, material aid, advice, positive feedback, physical assistance and social participation. However, more than 50 % of families indicated the need for more social support, suggesting an unmet need for help in coping. Second, the sample size was less than planned because many individuals did not complete the second set of questionnaires. Sample calculations are meant only to provide a target based on the a priori hypothesis on the anticipated effect size. After the study is complete, the actual results with 95 % confidence intervals are used to determine whether enough patients have enrolled for this study. The observed differences in the pre/post comparisons were small and the confidence interval on the pre/post differences did not include the large effect sizes, and thus the number of enrolled patients is sufficient to conclude no benefit from simple exposure to the website. Third, the before and after study design cannot provide conclusive findings as to the benefits of the website. However, the pre/post design is, if anything, generally biased toward a larger and positive effect.

In conclusion, healthcare providers often have limited time to spend with scoliosis patients who are considering surgery and their families. The results of this study found that simply providing access to an evidence-based website can increase the knowledge of patients and parents, but despite the promise of e-health, it had no impact on their coping and perceptions of social support. An e-health strategy may be used to increase knowledge, but enhancement of coping will continue to need direct clinical intervention.
